# Bringing value to cancer research

**DOI:** 10.3389/fonc.2025.1580575

**Published:** 2025-05-15

**Authors:** Casey J. Allen, Horn M. Danea, Fabrice Smieliauskas, Stephen Edge, Rachel A. Greenup

**Affiliations:** ^1^ Division of Surgical Oncology, Institute of Surgery, Allegheny Health Network, Pittsburgh, PA, United States; ^2^ School of Pharmacy, University of California, San Francisco, San Francisco, CA, United States; ^3^ Department of Economics and Department of Pharmacy Practice, Wayne State University, Detroit, MI, United States; ^4^ Roswell Park Comprehensive Cancer Center and Jacobs School of Medicine, University at Buffalo, Buffalo, NY, United States; ^5^ Department of Surgery and Smilow Cancer Hospital, Yale School of Medicine, New Haven, CT, United States

**Keywords:** quality, cost, shared decision-making, goal-concordant care, clinical trials

## Abstract

This article argues for incorporating a broader definition of “value” into cancer clinical trials. Current trials primarily focus on efficacy and safety, neglecting patient-reported outcomes (PROs) such as quality of life, financial toxicity, and time burden, as well as cost-effectiveness. We propose a novel framework integrating oncologic outcomes, PROs, and cost analyses. We also propose a multidimensional visual tool, such as a radar chart, to facilitate better-informed, value-based shared decision-making. This requires a collaborative approach, involving stakeholders in defining value metrics. While acknowledging challenges such as increased administrative burden and data interpretation complexities, a comprehensive framework can substantially improve patient-centered cancer care. The ultimate goal is to standardize value assessment in cancer research, leading to more equitable and effective care.

## Introduction

Extraordinary costs and waste have prompted the United States healthcare sector to shift from promoting high-volume care to a system based on value (1). Traditionally, “value” was defined as juxtaposing health outcomes against the direct financial costs of achieving those outcomes (2–4), however the concept of “value” continues to evolve to incorporate various domains that matter to patients, caregivers, and the healthcare ecosystem (5–7).

Clinical trials have traditionally focused on evaluating the efficacy and safety of interventions, neglecting crucial aspects of value. In oncology, this narrow scope overlooks the broader impact of cancer treatment on patients’ lives, including financial toxicity, time commitment, and quality of life (QOL) (8, 9). These burdens can lead patients to forgo treatments, especially when high-value care across multiple domains is not clearly defined (10). The inclusion of a more holistic, broader set of value measures in cancer clinical trials will empower patients and clinicians to make informed treatment decisions.

Despite the creation of several value frameworks (3, 5, 6), health systems and research communities remain challenged to address broader definitions of value (11). The American Society of Clinical Oncology developed a value assessment tool termed the Net Health Benefit that numerically scores treatments based on clinical advantages, side effects, QOL, and costs (5). The National Comprehensive Cancer Network endorses a visual tool providing a consensus-driven rating of various treatments in its “Evidence Blocks” that address efficacy, safety, and cost (6). These varied frameworks fail to provide a widely accepted measurement standard.

The National Cancer Institute National Clinical Trials Network (NCTN) develops and executes cancer clinical trials administered through Canadian and United States cooperative groups including the Alliance for Clinical Trials in Oncology (Alliance). NCTN groups rigorously scrutinize the safety and effectiveness of various oncology interventions. These groups strive to inform applications of novel therapeutics, guideline development, and healthcare delivery modalities. Thus, NCTN cooperative groups are poised to drive high-value cancer care.

## Understanding the need

Creating a new paradigm of value requires integrating complex, multidimensional concepts that accurately capture relationships between outcomes and costs, including both direct financial expenses and indirect repercussions such as effects on employment, time, and caregiver strain. As strides are made toward a cohesive measurement of value in cancer care, a discernible difference remains in the priorities assigned to value metrics (11). As value evolves within clinical trials and cancer care delivery, there is a critical need to rebuild a framework to incorporate the diverse perspectives of various stakeholders.

Recognizing this need, the Alliance Value in Cancer Care (ViCC) subcommittee surveyed Alliance members to identify the priorities of their clinicians, researchers, and patient advocates (12). Regardless of professional and advocacy subgroup, respondents prioritized patient-reported outcomes above traditional oncologic outcomes (12).

## A framework for value in oncology research

How can we best conduct research to determine treatments that optimize both patient priorities and healthcare resources? This issue, however, presents an opportunity to incorporate a richer array of measures that reflect a societal perspective on cancer care. This includes assessing QOL, personal financial impact, time engaged in the healthcare system, and other patient-prioritized domains. Including these endpoints in clinical research will create a more holistic view of outcomes than is seen in existing value frameworks (11).

To that end, the Alliance ViCC group prioritized the development of a framework that incorporates key value measures into clinical trial design. The Framework for Value in Oncology Research (FAVOR) provides a framework that supports researchers throughout the phases of clinical trial design, data collection, and outcome evaluation. We envision that this will promote a collaborative environment, encouraging researchers to work with stakeholders to integrate value measures into their studies.

We propose routine capture of certain key disease-agnostic domains into clinical trials:

### Oncologic outcomes

Fundamental metrics in cancer trials, including overall survival (OS), recurrence-free survival (RFS), and progression-free survival (PFS), that are essential for evaluating treatment effectiveness.

### Patient-reported outcomes

Tools that collect data from patients and caregivers on health status, QOL, and symptom burden.

### Costs and cost-effectiveness

Direct financial and societal costs and cost-effectiveness using measures including quality-adjusted life year (QALY) and equal value of Life Years (evLY) to assess value from healthcare, societal, and payer perspectives.

### Costs to the patient and caregivers

#### Direct patient and caregiver costs

Out-of-pocket medical costs, travel costs, and informal care costs are borne by patients, their families, and caregivers.

#### Financial toxicity

Financial distress experienced by patients during treatment, including from costs due to lost productivity (13, 14).

#### Time toxicity

The significant time patients/caregivers spend on healthcare (15).

Notably, existing cooperative group trials have incorporated some of these domains as secondary endpoints.

One example is the OptimICE-pCR Alliance trial (NCT05812807) that tests the effects of de-escalation of adjuvant immunotherapy for triple-negative breast cancer in patients who achieved a pathological complete response to neoadjuvant therapy (16). Endpoints include healthcare costs, cost-effectiveness, and several elements of costs to patients and their caregivers. An analysis of the Cancer Clinical Trials Group CO.17 (NCT00079066) trial demonstrated the feasibility of evaluating time toxicity. Patients receiving infusion treatment experienced fewer “home days” (days with no physical healthcare system contact) compared to patients receiving supportive care alone (17). These trials have demonstrated the feasibility of incorporating value domains into clinical research, potentially influencing clinical practice by providing a more comprehensive understanding of treatment impacts beyond traditional metrics.

### Patient-centered value: an example

**Figure 1:**
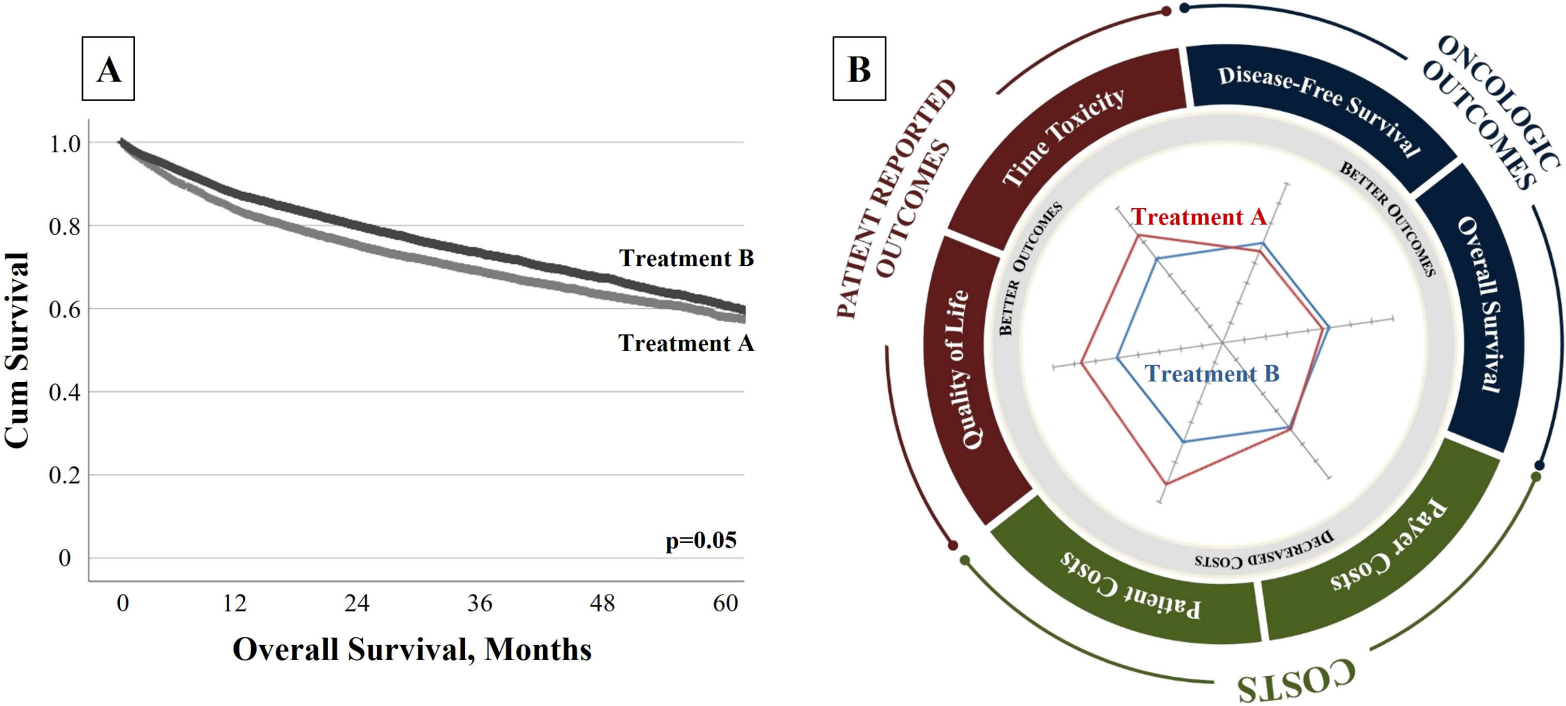
**(A)** Traditional Single-Dimensional Clinical Trial Outcome. “Treatment B” is a novel therapy that improves survival when compared to “Treatment A”, but these benefits are marginal. **(B)** Radar Chart to Communicate FAVOR Value Framework. A radar chart allows the user to assess multiple metrics simultaneously and affirms the multidimensional aspect of “value.” Here, the chart depicts the relative performance of two theoretical treatments, assessing both traditional oncologic metrics and patient-centric and cost measures. Although “Treatment B” provides a marginal survival advantage, “Treatment A” offers significant improvements in patient-centered and cost measures. This comprehensive assessment may facilitate more confident and informed value-based decisions.

To illustrate how this concept may influence decisions, consider an example of two theoretical treatments: “Treatment A” and “Treatment B.” Treatment B is a novel therapy that improves survival compared to Treatment A, but its benefits, although statistically significant, are clinically marginal (**Figure 1A**). It might be inferred that Treatment B is the appropriate choice for all patients due to its improvement in survival. But if Treatment A provides better QOL and lower costs of care, which treatment provides higher “value”?

With an expanded framework, patients and clinicians could be better informed. To help communicate these data, a radar chart depicts quantitative variables on multiple axes stemming from a single point to provide a clear representation of the multifaceted value dimensions (**Figure 1B**). The tool shows that while Treatment B has a marginal improvement in survival, Treatment A provides superior QOL and less treatment toxicity at lower costs. With this information, patients and providers can make better-informed decisions based on the priorities of care. Some patients might opt for the treatment that provides survival benefit, regardless of toxicities and costs (Treatment B), while others might prioritize their well-being at the potential expense of longevity (Treatment A). This approach is an effective way to communicate to stakeholders the trade-offs of treatment options in shared decision-making. It also allows stakeholders to focus on outcomes most pertinent to them. Radar charts not only stand as practical tools to foster shared decision-making but also serve to potentially guide clinical trial development—providing a snapshot to aid in value assessment (18). This framework may help not only patients and providers but also payers and policymakers select the optimal approaches on a population level.

This example demonstrates that solely measuring conventional outcomes deprives patients and providers of crucial information, resulting in suboptimal treatment decisions. While this information can be assessed through *ad hoc* or anecdotal methods, we should expand the gold standard of research (the clinical trial) to encompass domains of care that reflect multiple treatment outcomes and costs.

## Discussion

The steps involved include the development and adoption of a framework for oncology research. To pragmatically employ this framework, it will require reviews of published trials with diverse endpoints, feedback from patients and caregivers receiving care outside of a clinical trial setting to ensure their priorities are addressed, and a demonstration of the framework’s applicability in clinical trial design and its utility in improving communication around therapeutic benefits and risks. The ultimate goal is to increase trial inclusion and the reporting of value-based domains. By adopting a collaborative approach, this initiative facilitates the integration of standardized value assessments into research studies, the implementation of educational programs to enhance the understanding of value-based care, and advocacy for standardized reporting methods.

Anticipated barriers include addressing new complexities in trial design, managing the additional administrative burden and costs associated with more complex data collection, facing analytical challenges in interpreting the data, securing funding, and effectively communicating value to stakeholders. Patients, caregivers, providers, private and government payers, health administrators, and industry and government sponsors may have competing motivations across trial design and enrollment (10), complicating how value is incorporated and measured. Special attention must be paid to the diversity of priorities across heterogeneous patient populations, which are often not reflected in clinical trial participants. There will be questions of using measures specific to one cancer type or mode of treatment, or measures that address common issues across all cancer types.

Furthermore, clinical trials serve the primary purpose of evaluating the efficacy and safety of interventions. While value, encompassing societal and patient cost considerations, is undoubtedly crucial, it is contingent upon external factors such as manufacturer pricing, patent laws, insurance coverage, and cost-sharing. To study the cost domain within clinical trials, where the exact cost of a drug may not yet be determined, researchers can use cost simulations based on historical data or analogous therapies.

Determining whether these assessments should be incorporated into all trials, post-registration trials, or separate real-world efficacy trials requires consideration. However, the framework may be particularly applicable in post-market trials, where drug costs are more predictable, as well as in early-phase trials where cost simulations can be used based on analogous therapies. This approach allows for a more realistic assessment of value, even before the final pricing is established. Additionally, patient experience costs, such as travel and parking, may differ between a clinical trial setting and the standard of care. To account for these differences, we propose using adjusted models that account for the differences, ensuring that the assessments remain relevant in both settings.

Ultimately, the goal is to support investigators in integrating standardized value assessments into their research studies. As we strive to develop a novel value-based framework for cancer care, it will be necessary to draw upon insights from existing trials. Additionally, feedback from patients and caregivers receiving care outside the clinical trial setting is crucial to ensure the inclusion of contemporary cancer care aspects not covered by existing tools. Approaching value from a broader perspective offers distinct advantages for treatment decision-making and the provision of patient-centered care.

## Data Availability

The original contributions presented in the study are included in the article/supplementary material. Further inquiries can be directed to the corresponding author.
